# Rapid and Efficient Purification of Functional Collectin-12 and Its Opsonic Activity against Fungal Pathogens

**DOI:** 10.1155/2019/9164202

**Published:** 2019-07-29

**Authors:** Jie Zhang, Anna Li, Chang-qing Yang, Peter Garred, Ying Jie Ma

**Affiliations:** ^1^The Laboratory of Molecular Medicine, Department of Clinical Immunology, Section 7631, Rigshospitalet, Faculty of Health and Medical Sciences, University of Copenhagen, Ole Maaloesvej 26, 2200 Copenhagen N, Denmark; ^2^Department of Clinical Pharmacy, School of Basic Medicine and Clinical Pharmacy, China Pharmaceutical University, 211198 Nanjing, China

## Abstract

Collectin-12 (collectin placenta 1, CL-P1, or CL-12) is a newly identified pattern recognition molecule of the innate immune system. Recent evidences show that CL-12 plays important roles not only in innate immune protection against certain clinically important pathogens but also in scavenging of host molecules, leukocyte recruitment, and cancer metastasis. Furthermore, CL-12 has been shown to be associated with the pathogenesis of human diseases such as Alzheimer's disease and multiple sclerosis lesion development. Therefore, the functional consequence of CL-12 remains intriguing and awaits further elucidation. However, available protocols for the purification of recombinant CL-12 with high purity are laborious and inefficient and hamper further functional studies. Here, we report a simple, rapid, and efficient solution to obtain biologically active CL-12 with high purity. We established stable transfected Flp-In™-CHO cells expressing the recombinant CL-12 extracellular domain in high amounts. Recombinant CL-12 was purified from cell culture supernatants using a 3-step rapid purification procedure utilizing disposable affinity and ion exchange minicolumns. Purified recombinant CL-12 adopted an oligomeric structure with monomers, dimers, and trimers and retained its binding capacity towards the *A. fumigatus* strain that has been described before. Furthermore, we demonstrated the opsonic properties towards eight clinical isolates of *A. fumigatus* strains and diverse clinically important fungal pathogens. Purified recombinant CL-12 revealed a differential binding capacity towards selected fungal pathogens in vitro. In conclusion, we demonstrate a rapid and efficient purification solution for further biochemical and functional characterization of CL-12 and reveal opsonic properties of CL-12 towards diverse fungal pathogens.

## 1. Introduction

Collectin-12 (CL-12), also known as collectin placenta 1 (CL-P1), is a pattern recognition molecule (PRM) of the innate immune system, which is expressed from the *COLEC12* gene located on chromosome 18p11.32 [1]. CL-12 was originally defined as a scavenger receptor C-type lectin because it shares structural and functional similarities with the type A scavenger receptor and collectins [2, 3]. CL-12 is mainly expressed in cells that originate from the endothelium, placenta, gastric stroma, and macrophage [4, 5]. As a cell membrane-bound scavenger receptor C-type lectin, CL-12 has been recently shown to possess three functional domains: the collagen-like domain (CD) that mainly recognizes negatively charged ligands, the carbohydrate recognition domain (CRD) that specifically binds some carbohydrate moieties on microbial and fungal cell walls, and the coiled-coil domain which additionally interacts with the modified low-density lipoprotein (LDL) [6]. Upon support of those unique structural features, CL-12 not only exhibits innate immune defense characteristic of opsonization and phagocytosis against certain bacteria and fungi [3, 7] but also reveals a scavenging property against damage-associated molecular pattern (DAMP), for instance, oxidized LDL [2–4]. CL-12 is also suggested to be involved in leukocyte recruitment and cancer metastasis through interaction with LewisX trisaccharide [8, 9]. Furthermore, CL-12 binds fibrillar *β*-amyloid protein, implying its important roles in the scavenging or pathogenesis of Alzheimer's disease [10]. A more recent finding also indicates that CL-12 is involved in myelin internalization by central nervous system- (CNS-) resident phagocytes, and it likely plays a role in multiple sclerosis lesion development [11]. The importance of CL-12 as the scavenger receptor C-type lectin has also been recently suggested through its potential involvement in *H. pylori*- (Hp-) gastric stromal cell (GSC) interaction, thus mediating the crosstalk between GSCs and dendritic cells to link innate and adaptive immunity [5]. Furthermore, CL-12 has been shown to play a role in complement activation not only through the classical pathway (CP) via association with C-reactive protein (CRP) and C1q but also through the alternative pathway (AP) via interaction with properdin [7].

Nevertheless, a soluble form of CL-12 has been recently detected in the supernatants of *in vitro* cultured cells expressing transmembrane recombinant CL-12 as well as human umbilical cord plasma [1]. Study using a soluble chimera of recombinant CL-12 CRD-alkaline phosphatase expressed in HEK293/EBNA-1 cells has previously shown that the CRD of CL-12 reveals binding specificity towards GalNAc and the tumor-associated antigen Tn antigen [12]. The soluble CL-12 without the transmembrane domain has also been recently shown to mediate the AP of complement activation upon opsonization of certain opportunistic fungal pathogens, for instance, *Aspergillus fumigatus* (*A. fumigatus*) [1].

The functional consequences of CL-12 remain intriguing questions that await further elucidation. A detailed investigation of the CL-12 function in innate and/or adaptive immunity requires purified protein. Isolation of native CL-12 from natural sources often remains difficult due to its low expression level in the restricted human sources. Recombinant protein expression offers flexibility and facilitates subsequent purification through a modification of recombinant protein with a fusion tag. However, available protocols for the purification of recombinant CL-12 with high purity are laborious and inefficient, requiring additional processing of the starting materials (e.g., polyethylene glycol precipitation or dialysis) and multiple steps of FPLC chromatography purification. The present study was designed to establish a recombinant protein expression system highly expressing recombinant human CL-12 extracellular domain and to provide a subsequent solution for the rapid and efficient purification of CL-12. In addition, the aim of the present study is to investigate the opsonic properties of CL-12 towards diverse clinically relevant fungal pathogens.

## 2. Materials and Methods

### 2.1. Production of Recombinant CL-12

Recombinant CL-12 extracellular domain (rCL-12ED) was produced using the Flp-In™ System (Invitrogen) as previously described [1]. In brief, cDNA encoding rCL-12ED (Lys60~Leu742) tagged with N-terminal 6 × histidine was designed into the Flp-In™ expression vector (pcDNA5/FRT) containing the Hygromycin B resistance gene. Stably growing Flp-In™-CHO cells containing the lacZ-Zeocin™ fusion gene were cotransfected with a mixture of Lipofectamine, the Flp recombinase expression plasmid pOG44, and the pcDNA5/FRT vector containing the *COLEC12* gene. As a negative control, the pcDNA5/FRT vector lacking the *COLEC12* gene was applied in parallel. The cells were cultivated in Ham's F-12 medium (Thermo Fisher Scientific, UK) supplemented by L-glutamine (2 mM) (Thermo Fisher Scientific, UK), penicillin/streptomycin solution Hybri-Max (1%) (Sigma-Aldrich), and heat-inactivated fetal bovine serum (10%) (Gibco) for 2 days in a humidified 37°C incubator with 5% CO_2_. Stable transfectants were then selected by cultivation in the medium containing Hygromycin (500 *μ*g/ml) (Invitrogen). When the culture flasks were 80~90% confluent, the supernatants were harvested and analyzed for the level of CL-12 expression by western blot or ELISA as described elsewhere.

### 2.2. SDS-PAGE and Western Blot

Proteins were separated by 4~12% SDS-PAGE gel under reducing or nonreducing conditions according to the method of Laemmli [13] and visualized by Coomassie Brilliant Blue or western blot as described previously [14, 15]. For western blot analysis of CL-12, the samples were subjected to SDS-PAGE, and CL-12 was detected with goat anti-human CL-12 polyclonal antibody (CL-12 pAb) (R&D Systems, USA) and HRP-conjugated donkey anti-goat IgG (H&L; GenScript, USA) or directly with HRP-conjugated mouse anti-His monoclonal antibody (anti-His mAb) (GenScript, USA).

### 2.3. *Aspergillus* Strains and Preparation of Conidia

Isolates of *A. fumigatus* strains were obtained from fatal clinical specimens (no. 7096, no. 6285, no. 6595, no. 6871, no. 6783, no. 6480, no. 6458, and no. 6648) of invasive pulmonary aspergillosis (IPA) (a kind gift from Professor Luigina Romani, Microbiology Section, Department of Experimental Medicine and Biochemical Science, University of Perugia). Resting conidia were prepared as previously described [1]. In brief, the *A. fumigatus* clinical isolates were grown on a Sabouraud dextrose agar supplemented with chloramphenicol by agar streak for 4 days at 28°C. Abundant conidia were obtained under these conditions. The conidia were harvested by washing the slant culture with PBS supplemented with 0.025% Tween 20 (PBS-T) and gently scraping the conidia from the mycelium with sterile cotton-tipped applicators. The conidia were then allowed to settle by gravity, followed by filtration through a sterile 40 *μ*M cell strainer to remove hyphal fragments and cell debris. After extensive washing with PBS-T, the conidia were counted and diluted to the desired concentrations. The *A. fumigatus* conidia were heat inactivated at 121°C for 15 min prior to use.

### 2.4. Fungal Pathogens

Isolates of *Aspergillus* species and other fungal pathogens were obtained from clinical specimens (Innsbruck Medical University, Austria). The following strains were tested: no. 1—*Aspergillus fumigatus* AF293; no. 2—*Aspergillus fumigatus* 6871; no. 3—*Aspergillus terreus*, resistant to amphotericin C; no. 4—*Aspergillus terreus*, sensitive to amphotericin C; no. 5—*Aspergillus flavus* 1598; no. 6—*Aspergillus niger* N32; no. 7—*Lichtheimia corymbifera* AS41; no. 8—*Mucor circinelloides* MAL-D3; no. 9—*Rhizopus arrhizus* 44-12; and no. 10—*Candida albicans*. The fungi were heat inactivated as depicted previously prior to use.

### 2.5. Protein Purification

Culture supernatants were harvested from the Flp-In™-CHO cell line stably expressing rCL-12ED, centrifuged for 30 min at 3000 rpm to obtain cleared supernatants, and then stored at 4°C as crude materials until use.

The supernatants were loaded onto HisPur Ni-NTA Magnetic Beads (Thermo Fisher Scientific, USA) preequilibrated with equilibration (EQ) buffer (30 mM imidazole in PBS/0.05% Tween-20, pH 8.0). After shaking incubation at 4°C for 1 hr, the bead was washed twice with wash buffer (50 mM imidazole in PBS/0.05% Tween-20, pH 8.0). Bound proteins were then eluted with elution buffer (250 mM imidazole in PBS, pH 8.0). A magnetic stand was utilized to collect the beads in all steps.

To perform further purification, the eluate was loaded onto a disposable desalting spin column (40K MWCO) (Zeba, Thermo Fisher Scientific, USA) preequilibrated with EQ/wash buffer (25 mM Tris-HCl, pH 8.0) and the flow-through was retained by microcentrifuge (1000 × g, 3 min). To further remove the impurities, the desalted flow-through was subjected to a disposable anion exchange spin column (Pierce, Thermo Fisher Scientific, UK) preequilibrated with EQ/wash buffer. After thorough washing, the spin column was eluted with an increasing concentration of NaCl (0~0.5 M) in EQ/wash buffer by microcentrifuge (2000 × g, 5 min). The fractions containing the CL-12 band were pooled. The purification of CL-12 was monitored by SDS-PAGE and western blot analysis. The pooled fractions were eventually desalted against sterile PBS using a desalting spin column as previously described for further analysis. The purity and oligomeric pattern of CL-12 were determined by SDS-PAGE and western blot analysis.

### 2.6. ELISA

The CL-12 level in the culture supernatants and purified samples were measured as described previously. In brief, microtiter plates were coated with goat anti-human CL-12 polyclonal antibody (R&D Systems, USA) in PBS. After washing with PBS containing 0.05% Tween 20 (PBS-T), the plates were then incubated with the samples at room temperature for 3 h with agitation. Bound proteins were detected with a mixture of mouse anti-human CL-12 mAbs that was produced in our laboratory, followed by incubation of HRP-conjugated goat anti-mouse IgG (Dako). Final peroxidase reaction was visualized by TMB ONE (Kem-En-Tec Diagnostics A/S, DK). The CL-12 level in the samples was calculated based on the standard curve fitted with commercial recombinant CL-12 (R&D Systems, USA).

### 2.7. Recognition of Fungal Pathogens by Recombinant CL-12

The binding of soluble CL-12 to *Aspergillus fumigatus* (*A. fumigatus*) (clinical isolate 6871, University of Perugia, Italia) was performed as described previously [1, 14, 16]. In brief, clinical isolates of *A. fumigatus* strains were incubated with purified CL-12 (3.25~26 *μ*g/ml) at 37°C for 1 hr. Bound proteins were detected with CL-12 pAb and Alexa Fluor 488-conjugated donkey anti-goat IgG (Invitrogen, USA) and finally analyzed with a flow cytometer (Gallios, Beckman Coulter) or fluorescence microscopy (EVOS FL Cell Imaging System, Life Technologies). In some experiments, a panel of diverse fungal pathogens was screened for CL-12 binding as described above.

### 2.8. Complement Activation Assay

Complement C3b and terminal complement complex (TCC) deposition was determined as previously described [1]. In brief, *A. fumigatus* was preincubated with rCL-12ED (6.5 *μ*g/ml) before the incubation of MBL-deficient serum (serum^MBL-^) (10%) in the presence of EGTA-Mg^2+^. After washing, C3b and TCC deposition was detected by rabbit anti-human C3c pAb (Dako)/FITC-linked swine anti-rabbit IgG (Dako) or mouse anti-human C5b-9 mAb (BioPorto Diagnostics A/S, Denmark)/FITC-linked goat anti-mouse IgG (Dako), respectively, and analyzed by flow cytometry (Gallios, Beckman Coulter).

### 2.9. Statistical Analysis

Comparisons between groups were made by one-way ANOVA and GraphPad Prism, version 5.0 (GraphPad Software). *P* values less than 0.01 were considered significant.

## 3. Results

### 3.1. Production of Recombinant CL-12

**Figure 1:**
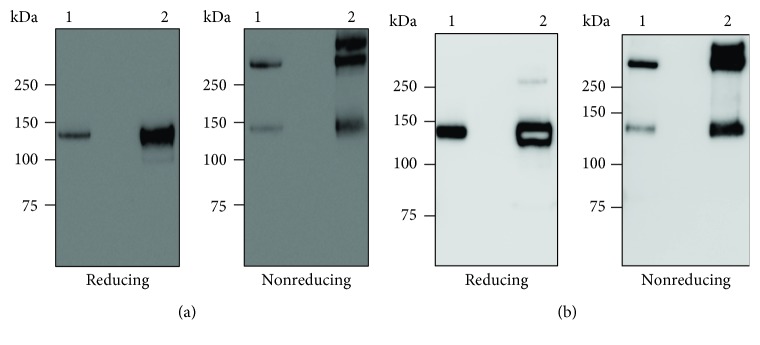
Expression of recombinant CL-12 in the Flp-In™-CHO cells. Supernatants were harvested from the cultivation of the Flp-In™-CHO cells expressing rCL-12ED. Culture supernatants were then subjected to 4-12% SDS-PAGE and analyzed with CL-12 pAb (a) or anti-His mAb (b) by western blot. Lane 1, rCL-12R&D; lane 2, culture supernatant.

N-terminally His-tagged recombinant CL-12 was produced through the establishment of a stable CHO cell line expressing a recombinant CL-12 extracellular domain (rCL-12ED) as described in Materials and Methods. The culture supernatants of rCL-12ED-expressing CHO cells were used to analyze soluble CL-12 expression in western blot. When the supernatants were analyzed with either CL-12 pAb or anti-His mAb as shown in Figures 1(a) and 1(b), the gel mobility patterns were mutually consistent. Under nonreducing conditions, rCL-12ED presented a multimeric assembly laddered with three bands corresponding to monomers, dimers, and trimers, while under reducing conditions it was restored to monomers with gel mobility corresponding to ~130 kDa (Figures 1(a) and 1(b)).

### 3.2. Purification of Recombinant CL-12

**Figure 2:**
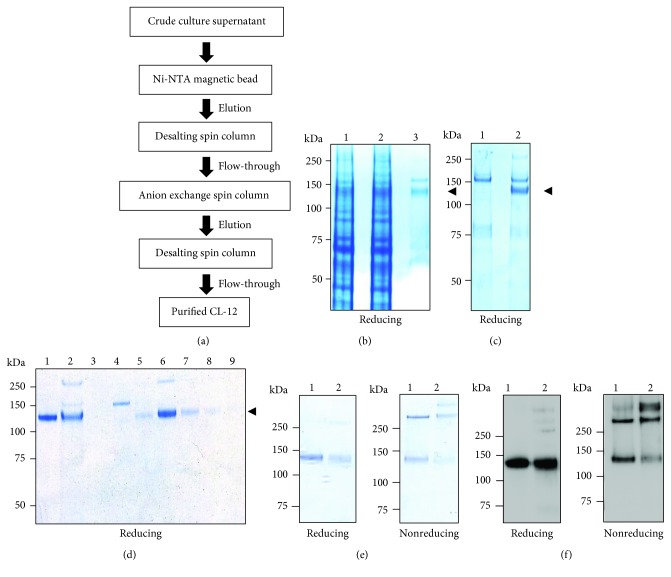
Purification of recombinant CL-12. (a) Illustrative diagram for CL-12 purification. (b) Purification of recombinant CL-12 by Ni-NTA magnetic beads. rCL-12ED-expressed culture supernatants were incubated with Ni-NTA magnetic beads, followed by washing with buffer containing 50 mM imidazole and elution with buffer containing 250 mM imidazole. Purification samples were analyzed by 4~12% SDS-PAGE. Lane 1, loading material; lane 2, flow-through; lane 3, eluate. (c) Determination of impurities in the eluate. Supernatants from the cultivation of CHO/control were conducted with the same procedure of purification. The eluate of beads incubated with supernatants of CHO/control cultivation (lane 1) or rCL-12ED-expressed CHO cultivation (lane 2) was analyzed by 4~12% SDS-PAGE. (d) Impurity removal by an anion exchange spin column. CL-12-containing eluates were run onto an anion exchange spin column after removal of imidazole by a desalting spin column. Bound proteins were fractionated with a NaCl gradient (0~0.5 M) and analyzed by 4~12% SDS-PAGE. (e) Analysis of purified CL-12. CL-12-containing fractions seen in panel (d) were pooled, desalted, and concentrated to the desired concentration. The purity and oligomeric pattern were then determined in SDS-PAGE (e) and western blot (f) analysis. Lane 1, rCL-12R&D; lane 2, purified rCL-12ED. Arrow indicates the major rCL-12ED band.

Recombinant CL-12 was isolated from the culture supernatants of rCL-12ED-expressing CHO cells using the following four-stage procedure as shown in Figure 2(a): (i) separation by Ni-NTA magnetic bead; (ii) desalting to remove imidazole; (iii) purification by the Mono Q spin column, and (iv) desalting for high salt removal. The purity of CL-12 was monitored during the purification procedure.

The culture supernatants were affinity separated by Ni-NTA magnetic beads, resulting in the specific capture of His-tagged CL-12 with an ~160 kDa contaminant (Figure 2(b)). To determine that the contaminant was derived from media compounds including CHO cell-expressed proteins that bound nonspecifically to magnetic beads, the supernatants harvested from mock-transfected CHO cells (CHO/control) were applied with the same procedure of separation. As shown in Figure 2(c), the ~160 kDa contaminant reappeared, indicating that nonspecific binding of the media compounds occurred during incubation with the magnetic beads. To eliminate the ~160 kDa contaminant, the eluates of the magnetic beads were further purified by the performance of the Mono Q spin column. The elution fractions containing CL-12 were pooled, desalted, and concentrated (Figure 2(d)). The concentration of purified CL-12 was measured by quantitative ELISA. The purity and oligomerization state were determined by SDS-PAGE and western blot analysis (Figures 2(e) and 2(f)). The purification scheme yielded recombinant CL-12 with considerable purity (~95%) and functional oligomer formation (Figures 2(a), 2(e), and 2(f)).

### 3.3. Biological Activity of Purified CL-12

**Figure 3:**
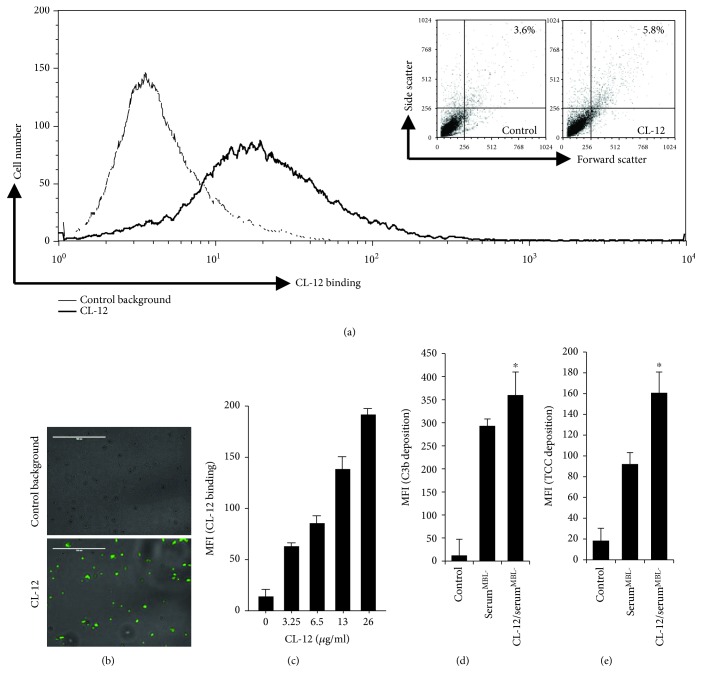
Functional analysis of purified CL-12. Binding of purified CL-12 (6.5 *μ*g/ml) to *A. fumigatus* was assessed by flow cytometry (a) or fluorescence microscopy (scale bar, 100 *μ*m; 40x magnification) (b). CL-12-induced agglutination was assessed by a change of forward and side scatter morphology and shown in the inset in (a). (c) Dose-dependent binding of purified CL-12 to *A. fumigatus*. (d, e) Amplification of complement activation by purified CL-12. *A. fumigatus* were incubated with purified CL-12 (6.5 *μ*g/ml) prior to inducing complement activation by serum^MBL-^ in the presence of 5 mM Mg^2+^/10 mM EGTA, and then C3b (d) or TCC (e) deposition was analyzed. The mean fluorescence intensity (MFI) was used to assess protein binding. The results are expressed as the mean ± SEM of at least three independent experiments. ^∗^*P* < 0.01.

We have previously demonstrated that soluble CL-12 is a pattern recognition molecule initiating the alternative pathway (AP) of complement activation [1]. To determine the biological activity of purified CL-12, we determined the activities in respect to opsonization and complement activation through the C3b and TCC deposition assay on *A. fumigatus* as established previously [1]. As shown in Figures 3(a)–3(c), purified CL-12 bound *A. fumigatus* in a dose-dependent manner and induced agglutination of *A. fumigatus* conidia. Upon opsonization, purified CL-12 significantly amplified both C3b and TCC deposition when serum^MBL-^ was used as a source of complement to induce complement activation (Figures 3(d) and 3(e)).

### 3.4. Characterization of CL-12 Binding to *A. fumigatus* Clinical Isolates

**Figure 4:**
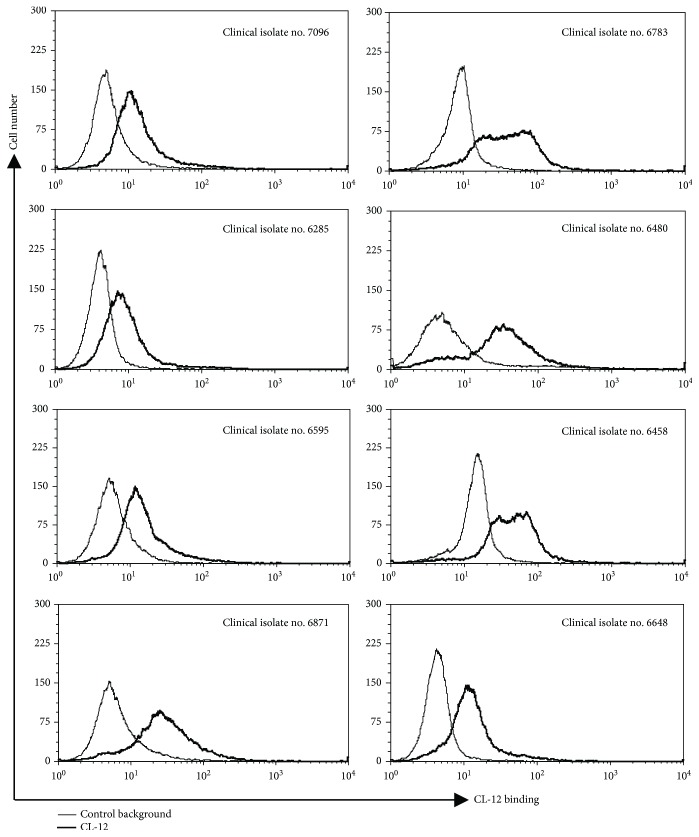
Opsonic properties of CL-12 towards *A. fumigatus* strains. Isolates of *A*. *fumigatus* strains were obtained from clinical specimens. Binding of purified CL-12 (6.5 *μ*g/ml) to the *A*. *fumigatus* strains was assessed by flow cytometry. The MFI was used to assess protein binding. Results are representative of at least three independent experiments.

*A. fumigatus* conidia from eight clinical isolates (no. 7096, no. 6285, no. 6595, no. 6871, no. 6783, no. 6480, no. 6458, and no. 6648) were incubated with purified CL-12. Bound CL-12 was detected by flow cytometry as described in Materials and Methods. Flow cytometry analysis showed that CL-12 recognized all the *A. fumigatus* conidia with stronger binding to the four conidia (no. 6871, no. 6783, no. 6480, and no. 6458) (Figure 4).

### 3.5. Characterization of CL-12 Binding to *Aspergillus* Species and Fungal Pathogens

**Figure 5:**
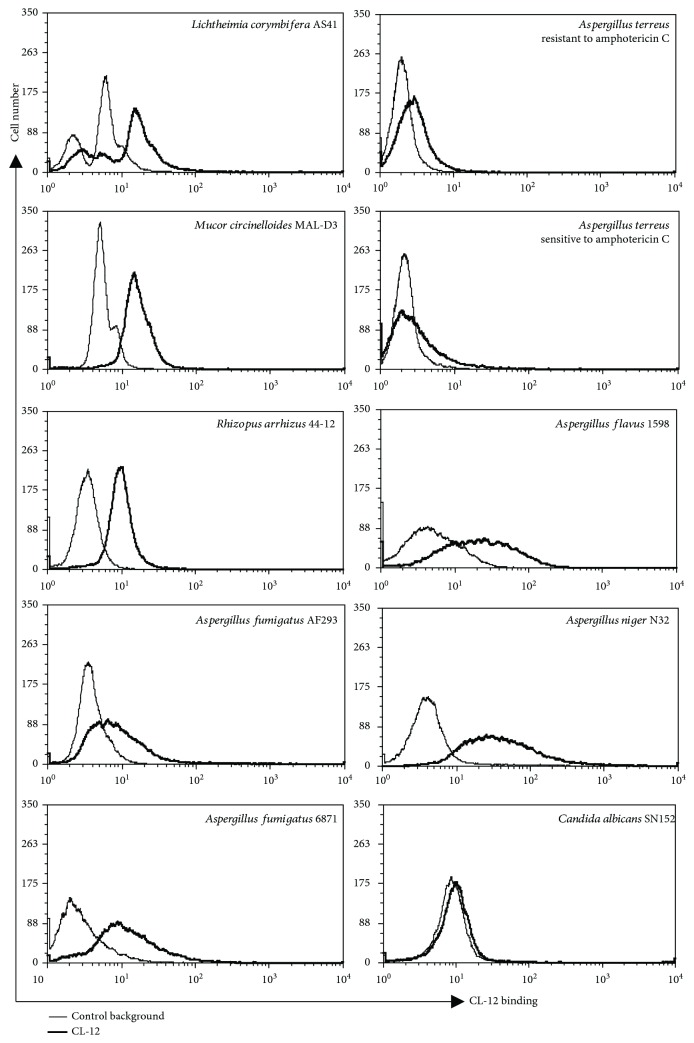
Opsonic properties of CL-12 towards the *Aspergillus* species and fungal pathogens. Isolates of fungal pathogen species were obtained from clinical specimens. Binding of purified CL-12 (6.5 *μ*g/ml) to a panel of different fungal pathogens was assessed by flow cytometry. The MFI was used to assess protein binding. Results are representative of at least three independent experiments.

Four different species of *Aspergillus conidia* (*A. fumigatus*, *A flavus*, *A. niger*, and *A. terreus* sensitive to amphotericin C and *A. terreus* resistant to amphotericin C) and four different fungal pathogens (*Lichtheimia corymbifera* AS41, *Mucor circinelloides* MAL-D3, *Rhizopus arrhizus* 44-12, and *Candida albicans* SN152) were tested as described above. Flow cytometry analysis showed that CL-12 is able to recognize all the fungal pathogens tested other than *Candida albicans* and *Aspergillus* species except for the *A. terreus* species sensitive to amphotericin C (Figure 5).

## 4. Discussion

CL-12, also known as CL-P1, is a newly identified novel collectin that plays an essential role in innate immunity [1, 2, 17]. However, accumulating evidences suggest that CL-12 might exert intriguing functions far beyond innate immune defense, for instance, scavenging of host molecules [3], leukocyte recruitment, and cancer metastasis [8, 9, 18], and also an influence in adaptive immunity [5]. Moreover, potential associations with the pathogenesis of diseases such as Alzheimer's disease [10], cancer [8], and multiple sclerosis [11] have also been revealed. Those intriguing implications of CL-12 highlights the need for a detailed investigation *in vivo* and *in vitro* and thus requires experimental tools for the preparation and purification of functional recombinant CL-12.

To get active recombinant protein for the further investigation of CL-12, we have established stably transfected Flp-In™-CHO cells highly expressing rCL-12ED. Determining the content of CL-12 in the culture supernatant, we found that rCL-12ED is mainly present as monomeric, dimeric, and trimeric structural scaffolds, and it was consistent with the pattern when a soluble form of CL-12 shed from *in vitro* cultured rCL-12 full length (Met1~Leu742)-expressing CHO cells was analyzed (data not shown). These results suggest that the oligomer assembly of CL-12 could also remain during the natural occurrence of a soluble form of CL-12 shed from cell membrane. Oligomerization of collectins dictates binding property and complement activation [19], thus this is of great importance for proper biological functions. Our results indicate that the rCL-12ED during secretion is highly oligomerized as expected to undertake the biological activities.

To purify recombinant CL-12 from the culture supernatant, we have developed a four-stage rapid purification method as described in the flow chart of the purification protocol and used the resulting protein to assess opsonic activity against fungal pathogens. The purification of His-tagged recombinant protein often employs an immobilized metal affinity chromatography (IMAC) approach employing agarose-conjugated nickel (Ni) or cobalt (Co) and requires many steps of processing of starting material (e.g., polyethylene glycol precipitation, buffer change, or harvest of material from serum-free media cultivation) and combined purification with gel filtration and affinity chromatography to increase final purity. Here, we present an alternative protocol for the purification of His-tagged recombinant CL-12 with high purity and functional activity. Our method for the isolation of recombinant CL-12 has the following advantages: (i) no requirement for processing steps of starting material, for instance, expression in serum-free condition to sidestep the complexity of purification, buffer change, concentration of material, or polyethylene glycol (PEG) precipitation, and (ii) a convenient and efficient purification procedure without the need for laborious processing. Using serum-containing medium, we were able to elevate the productivity of recombinant CL-12 during cultivation in contrast to cultivation in serum-free medium. Furthermore, using the crude culture supernatants as starting material without additional preparation, the four-step purification can be performed with only disposable beads and minicolumns and a magnetic stand and a table top microcentrifuge. The protocol enables the CL-12 purification to be finished within ~3.5 hr with high purity. In contrast to other existing methods involving PEG precipitation and/or FPLC/HPLC, our purification procedure can be efficient and useful in producing recombinant CL-12 for research laboratories with only standard cell culture equipment. Our results demonstrated that the purified CL-12 retains its oligomer assembly as required for proper biological functions of CL-12.

Invasive aspergillosis (IA) is an infection caused by the *Aspergillus* species and almost exclusively develops in immunocompromised patients with a high mortality rate. Inhalation of the *Aspergillus* conidia into the respiratory tract is the main path causing lung infection, which is hitherto the most frequent type of IA [20]. However, *Aspergillus* can infect different organs through the penetration of blood vessels, for instance, the heart, the liver, and the central nervous system (CNS) [20]. In general, *A. fumigatus* represents the most common cause of IA, followed by *A. terreus*, *A. flavus*, and *A. niger* [20, 21]. We have previously demonstrated that soluble CL-12 plays an important role as an integral part of the innate immune defense against *A. fumigatus* by opsonizing conidia, and we highlighted the role in complement amplification via the AP [1]. Our results substantiate that the purified CL-12 retains its functional activities including opsonization, agglutination, and complement amplification against *A. fumigatus*. Using the purified CL-12, we have been able to assess its opsonic activity against a variety of *A. fumigatus* clinical isolates and clinically important fungal pathogens including the *Aspergillus* species for the first time. Here, we observed that resting conidia (which represent the initial fungal spores inhaled into the lungs) from a variety of clinically important *Aspergillus* species (*A. fumigatus*, *A. flavus*, *A. niger*, and *A. terreus* resistant to amphotericin C) excluding *A. terreus* strains sensitive to amphotericin C are being recognized by the purified CL-12 with varied extent. Furthermore, we found that the purified CL-12 is also able to recognize other fungal pathogens, for instance, *Lichtheimia corymbifera* AS41, *Mucor circinelloides* MAL-D3, and *Rhizopus arrhizus* 44-12, but not *Candida albicans* SN152. These data suggest that CL-12 may play an important role in the innate immune defense against a wide spectrum of clinically important fungal pathogens by the opsonization of conidia and phagocytosis by professional phagocytes. As described in our previous findings, soluble CL-12 is capable of mediating complement amplification via the AP. Since the thick fungal cell wall blocks TCC formation and the subsequent cell lysis, C3b deposition of the fungal surface through soluble CL-12-mediated complement amplification is presumably also important to complement the associated weapon stimulating efficient phagocytosis or release of damaging compounds, oxidative burst, and killing by monocytes, bronchoalveolar macrophages, and polymorphonuclear cells. How membrane-anchored CL-12 is cleaved from the plasma membrane and what the biological fate is against a wide spectrum of fungal pathogens including the *Aspergillus* species are hitherto unknown and thus remain key questions awaiting elucidation in our further investigation.

In summary, we introduce a rapid and efficient protocol for the purification of recombinant CL-12, which enables the recovery of a preparation of active, oligomerized CL-12. Therefore, the protocol presented here could be applied to gain insight into the physiological and/or pathological roles of newly identified CL-12 for further studies. The opsonic properties towards clinically relevant fungal pathogens suggest that CL-12 is a pattern recognition molecule of high significance for innate immune defense against microorganisms with potential capabilities provoking a variety of innate immune effector mechanisms.

## Data Availability

The data used to support the findings of this study are available from the corresponding author upon request.
